# Comparison of modern high-speed vitrectomy systems and the advantages of using dual-bladed probes

**DOI:** 10.1186/s40942-020-00277-2

**Published:** 2021-01-19

**Authors:** R. Oravecz, D. Uthoff, N. Schrage, R. M. Dutescu

**Affiliations:** Aachen Centre of Technology Transfer in Ophthalmology (ACTO E.V.), An-Institute,, RWTH-University, Karlsburgweg 9, 52070 Aachen, Germany

**Keywords:** Vitreous, Vitrectomy, 20 gauge, 23 gauge, Vitreous model

## Abstract

**Purpose:**

This study analyzes the efficiency of different vitrectomy systems and compares single with double-bladed cutters.

**Methods:**

The systems EVA™ (DORC), Constellation^®^ Vision System (ALCON), megaTRON S4^HPS^ (Geuder) and Stellaris^®^ PC (Bausch and Lomb) were used. We chose 20G and 23G probes, since not all systems had switched to a smaller G at the time the study was conducted in 2016. Cut rates were varied in increments of 1000 cuts/min from 500 cpm to the system’s maximum and vacuum pressures were varied in increments of 100 mmHg, from 100 to 600 mmHg up to the individual system’s maximum. In this study water, egg white, Pluronic^®−^F127 gel and isolated porcine vitreous were used as models of human vitreous. The vitrectomy efficiency was calculated from the aspirated mass (g) within 30 s. The aperture of the different vitrectomy probes was filmed with a high-speed camera.

**Results:**

The area under the curve analysis showed differences in efficiency between vitrectomy systems. For water, a reverse relationship between the aspirated mass and cut rate was shown. By contrast, for most systems aspirated egg white and porcine vitreous showed a non-linear increase or decrease for 4000 cpm and above. For all vitreous surrogates, EVA™’s double-bladed probe aspirated significantly (p < 0.001) more vitreous than its mono-bladed probe. Video recordings showed less vitreous traction for double- in contrast to single-bladed probes.

**Conclusion:**

We can demonstrate differences in the efficiency of vitrectomy depending on the vitrectomy system used. Double-bladed probes were more efficient and probably safer than single-bladed probes.

## Key messages

- Despite the size, there are differences in the efficiency of vitrectomy depending on the vitrectomy system used.
- Double-bladed probes were more efficient in working up vitreous compared to single-bladed probes.
- Except for EVA™, high cut rates adversely affect vitreous removal.

## Introduction

The closed pars plana vitrectomy, established by Robert Machemer in 1971, has advanced to the most frequently applied therapy of vitreoretinal pathologies over recent decades [1]. These pathologies include vitreous hemorrhage, complicated retinal detachment, macular hole, intraocular foreign body or endophthalmitis [2–7]. Starting with 14- and 17-gauge single-port vitrectomy, O’Malley and Heintz then introduced the 20-gauge, three- port vitrectomy in 1975 [8]. Current microincision vitrectomy systems (MIVS) promote self-sealing sutureless sclerotomies, such as the 23-gauge sclerotomies developed by Eckardt, and the further minimization to 27-gauge by Oshima et al. [9, 10].

Aside from the further miniaturization of the scleral incision to mitigate trauma of the surgical procedure, a primary goal of recent vitreoretinal research is to increase the efficiency of the vitrectomy. To attain higher efficiency, one should bear in mind that vitreous removal is influenced by the sclerotomy size, the duty cycle of the vitrector (cut rate and aspiration), the infusion pressure and the aspiration vacuum. Regarding miniaturization, special attention needs to be devoted to the problem of flow in small diameter tubes.

In recent years high-speed vitrectomy systems with a cut rate of up to 10,000 cuts/minute (cpm) have become especially popular, due to higher cut rates causing decreased retinal traction [11]. Moreover, increasing the infusion pressure from around 30 mmHg in 20-gauge vitrectomy systems to 50 mmHg in 23- to 27-gauge systems allows for a sufficient flow rate through an ever-smaller diameter. Nevertheless, even with a maximum aspiration pressure, the aspiration rate in 25-gauge vitrectomy is lower than that of a 20-gauge system [12]. With respect to the duty cycle and movement of cutter blades, parabolic (pneumatic) and trapezoid (double pneumatic) cutter movements both have a decreased duty cycle at 1500 cpm compared to 600 cpm, although sinusoid (electric) duty cycles did not differ even at higher cut rates [13].

A novel development is the Ultravit High Speed Vitrectomy Probe (Constellation^®^ Vision System, Alcon) which operates the vitrector blades by bidirectional pulsed air in contrast to the current spring release mechanism [14, 15]. Furthermore, the Dutch Ophthalmic Research Center (DORC) (Zuidland, The Netherlands) introduced a vitrector with two angled blades, promising improved fluid dynamics and higher cut rates [16].

Regarding fluid control, the aspiration flow at the cutter orifice is difficult to control, as liquid removal depends not only on the vacuum pressure, but particularly on the viscosity of the fluids. This shift in viscosity between BSS and vitreous can cause unfavorable vitreoretinal tractions. This was particularly noticeable when using venturi pumps and peristaltic pumps, however, this risk has been reduced [12, 17].

This study analyzes the vitrectomy efficiency as a function of vacuum pressure and cut rates and the vitrectomy system as a whole, including the respective machine and cutter applied. In addition, high-speed videos of the vitrector during a duty cycle were recorded to differentiate the characteristics of each vitrectomy system. We chose 20 G and 23 G probes since not all vitrectomy systems offered smaller probes at the time the study was performed in 2016.

## Materials and methods

### Vitreous surrogates

Tap water, Pluronic^®^-F127 gel (Pluronic^®^-F127, Sigma-Aldrich, Germany), egg white and porcine vitreous were employed as substitutes of human vitreous. Pluronic^®^-F127 is a block-co-polymer of ethylene dioxide and propylene dioxide, and its viscosity is adjustable by varying its concentration and temperature.

The egg white was used within its shelf life. Eggs were stored at 4 °C, and the experiments were conducted at a room temperature of 21 °C. Porcine eyes were obtained from a local slaughterhouse in order to use their vitreous within 12 h of slaughter.

## Viscosity

Viscosity measurements were run as a pre-test by using rotation viscometry (Rotational Viscometer PCE-RVI 2, PCE Deutschland GmbH, Germany). The rotation viscometer measures the force needed to rotate a spindle submerged in the substance. Based on the electronically recorded twist moment measured in mPa s/cP, which depends on the geometry and rotational speed of the spindle, the viscosity can be measured in mPa s/cP (1 mPa s = 1 cP [centi-Poise]). Water has a viscosity of 1 mPa s at 20 °C. The spindle L2 (original from the company) was utilized for the tests. Moreover, for simulating human vitreous, an anti-lyotropic Pluronic gel (Pluronic^®−^F127, Sigma-Aldrich, Germany) was used in different concentrations. The states of aggregation are fluid (− 10–20 °C) or firm (22 − 37 °C), depending on the temperature. Consequently, the respective viscosity of Pluronic^®−^F127 gel at different concentrations was investigated as a function of temperature. Our aim was to find the particular concentration at room temperature with stable viscosity. In addition, the viscosities of the prepared porcine vitreous and egg white were measured under the same measuring conditions.

### Vitrectomy systems

For comparing the efficiency and characteristics of modern vitrectomy systems, the following systems were examined: Constellation^®^ Vision System (Alcon, Forth Worth, USA), EVA™ (Dutch Ophthalmic Research Center, DORC), Zuidland, The Netherlands), megaTRON S4^HPS^ (Geuder, Heidelberg, Germany), and Stellaris^®^ PC (Bausch&Lomb, New Jersey, USA).

The Constellation^®^ Vision System is a dual pneumatic drive technology vitrectomy machine with a Venturi vaccum pump. 20G and 23G Ultravit^®^ high-speed vitrectomy probes were applied. Of the three duty cycles to choose from in the Constellation^®^ Vision System (Port Biased Open for core-vitrectomy, Port Biased Closed), we chose the Port Biased Open, given its presumed higher vitrectomy efficiency. Moreover, the megaTRON S4^HPS^ and the Stellaris^®^ PC systems work with a Venturi vacuum pump as well. In contrast, the EVA™ system employs a peristaltic two-cylinder pump mechanism. For each system, the original cutters provided by the manufacturer were used. Additionally, the newly developed twin duty cycle (TDC) cutters in the EVA™ system and Mach2 in megaTRON S4^HPS^ systems were tested, all of which operate with double-bladed cutters.

Depending on the vitrectomy system, the maxima of the aspiration vacuum pressure and cut rates per minute (cpm) differ, as is summarized in Table 1.

**Table 1:** Range of cut rates and aspiration pressures for the vitrectomy systems, S4 (Geuder, Germany), Stellaris^®^ PC (Bausch & Lomb), Constellation^®^ Vision System (Alcon), EVA^™^ (DORC, The Netherlands)

	Stellaris PC Bausch & Lomb	Constellation vision system ALCON	Megatron S4 Geuder	Eva D.O.R.C.
Maximum Cut rate (cuts/min)	5000	5000 (20G), 7500 (23G)	2500 (20G), 6000 (23G)	8000
Maximum Aspiration Pressure (mmHg)	600	650	600	680

### Vitrectomy

All vitrectomy systems were evaluated using 20-gauge as well as 23-gauge vitrectomes submerged in water, 17.5% Pluronic^®−^F127 gel, egg white and porcine vitreous without infusion pressure in an open beaker. Starting at 500 cpm, the cut rates were increased stepwise (increments of 1000 cuts/min) until the maximum of the applied vitrectomy system was reached (5000 cuts/min for megaTRON S4^HPS^ and Constellation^®^ Vision System; 8000 cuts/min for EVA™). Starting at 100 mmHg, the aspiration pressure was increased in 100 mmHg steps until a maximum of 600 mmHg, except for in the cases of the EVA™ and Constellation^®^ Vision System (650 mmHg), was attained (see Table 1).

Vitreous surrogates were filed in borosilicate open beaker glasses centered on a microbalance (SBS-LW-2000A, Steinbers System, Germany). Vitrectomes were submerged in the test material, and vitrectomy was performed for each setting in triplicates for 30 s. The aspirated test material was measured in grams as weight loss by means of elimination of vitreous through the vitrectomy system.

### High-speed video recordings

To analyze the characteristics of the vitrectomes and their duty cycles, high-speed videos were recorded (CR3000 × 2 high-speed video-camera, Optronis, Germany). Therefore, anterior segments of porcine eyes were removed, and vitrectomies were filmed under illumination (Multied LT-V8-15, Optronics, Germany). For each vitrector, videos were recorded with a stepwise increase in vacuum pressures and cut rates according to the aforementioned protocol.

### Statistical analysis

To compare the mass aspiration of different vitrectomy systems and cutter designs for each vitreous surrogate, a two-tailed t-test was performed. For mono-bladed probes, the area under the curve analysis displays the aspirated mass against cut-rates from 0–6000 cpm at a fixed 600 mmHg aspiration pressure.

## Results

### Viscosity of vitreous surrogates

**Fig. 1:**
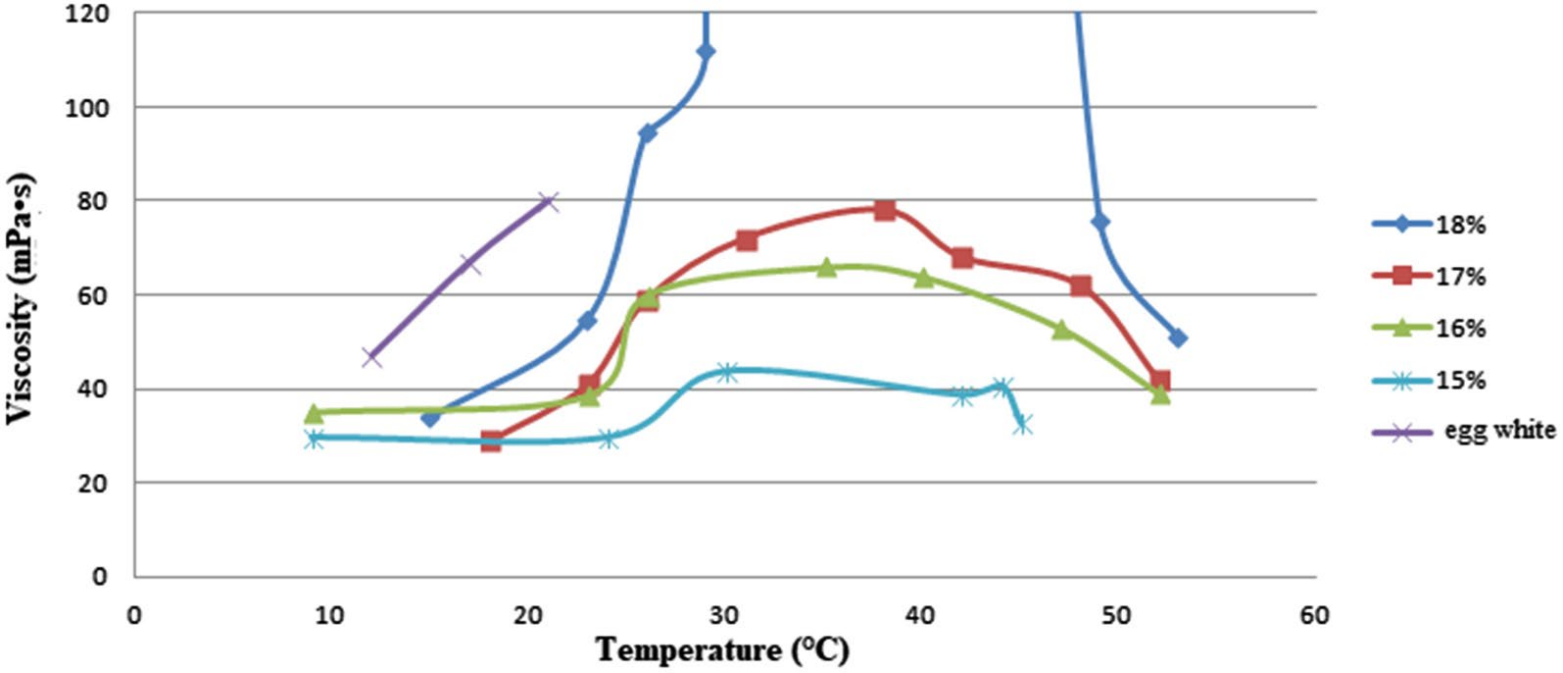
Viscosity (mPa s) of egg white and Pluronic F127^®^ in concentrations of 15% to 18% as a function of temperature (°C)

Water has a known viscosity of 1 mPa s, which has been confirmed in our tests. Pluronic^®−^F127 in concentrations of 15%, 17% and 18% revealed similar viscosities in a narrow range of 18–25 °C (Fig. 1). Pluronic^®−^F127 in a concentration of 17.5% and with an estimated viscosity of around 40 mPa s was used in our experiments. Higher viscosities like in porcine and human vitreous were not achieved, since a viscosity of even 18% Pluconic F127 did not show a plateau in viscosity at a certain temperature. On the other hand, concentrations above 18% were too viscous to be worked off by vitrectors.

The native porcine vitreous showed high variation in viscosity between 60–200 mPa s, which can be explained by its inhomogeneity based on its collagen and hyaluronic acid fundamental structure. The measurements of egg white also showed variable viscosity ranging from 45 mPa s to 80 mPa s.

### 20-gauge and 23-gauge Vitrectomy

#### Water

**Fig. 2:**
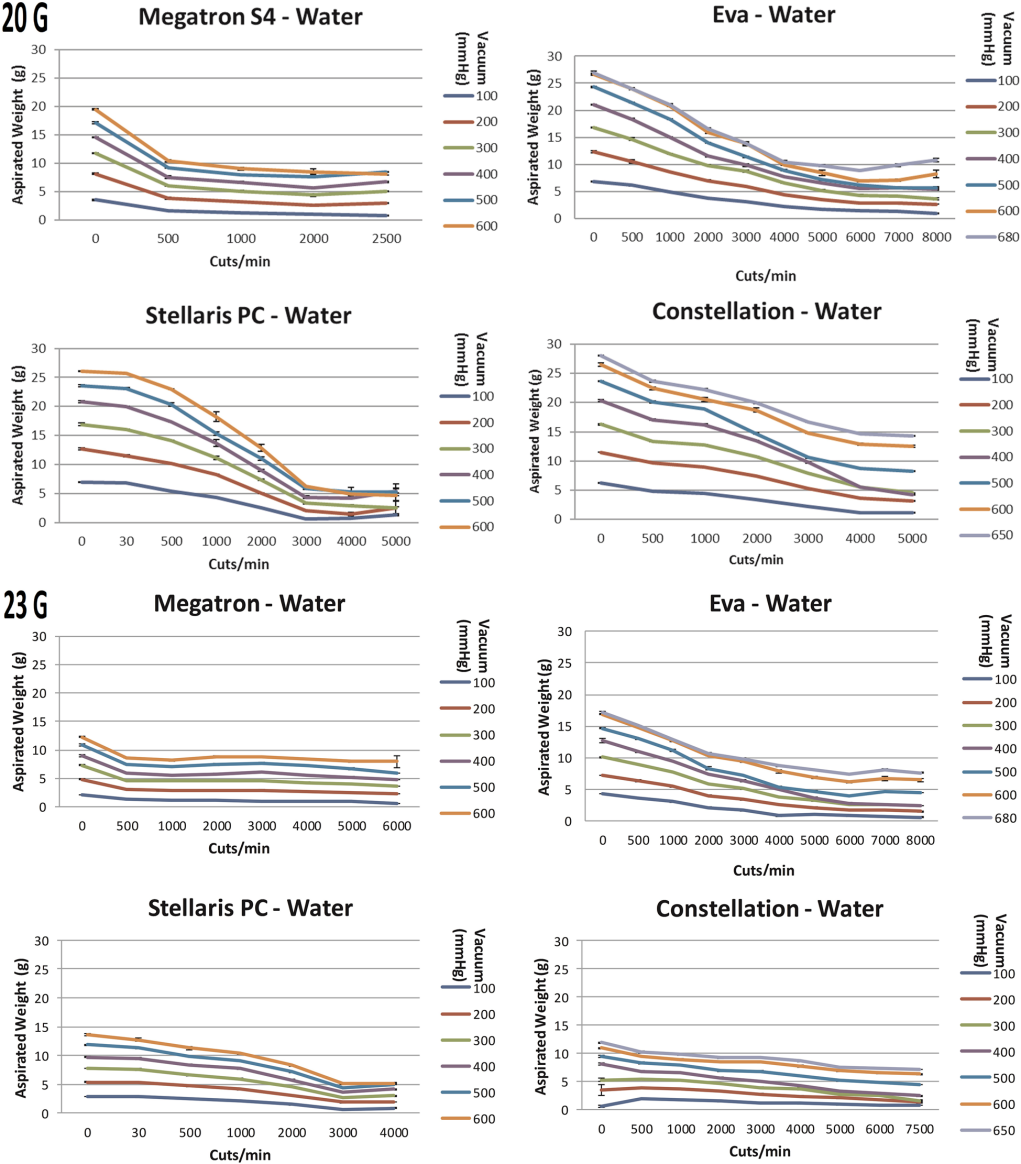
The aspiration of water (g) as a function of cut rate and vacuum pressure for 20- and 23-gauge vitrectomes for the vitrectomy systems Constellation^®^ Vision System, EVA^™^, megaTRON S4^HPS^ and Stellaris^®^ PC

For the vitrectomy of water most systems showed a significant decline in aspirated mass at high cut rates (Fig. 2) This is the case for most systems except for 20G/23G megaTRON S4^HPS^ probes, 23 G Constellation^®^ probes and both double-bladed probes of EVA™ and megaTRON S4^HPS^. Here, no significant differences (p > 0.05) were seen for high cut rates compared to 0 cpm. For EVA™, the aspirated mass of water was significantly lower at 6000 cpm compared to 0 cpm for both 20G (p < 0.0001) and 23G (p < 0.0001). Comparable results are shown for Stellaris^®^ PC (20G, p < 0.001, 23G, p < 0.05) for cut rates at and above 3000 cpm compared to 0 cpm. For Constellation^®^ 20G probes significantly less (p < 0.0001) mass was aspirated at 5000 cpm compared to 0 cpm.

**Fig. 3:**
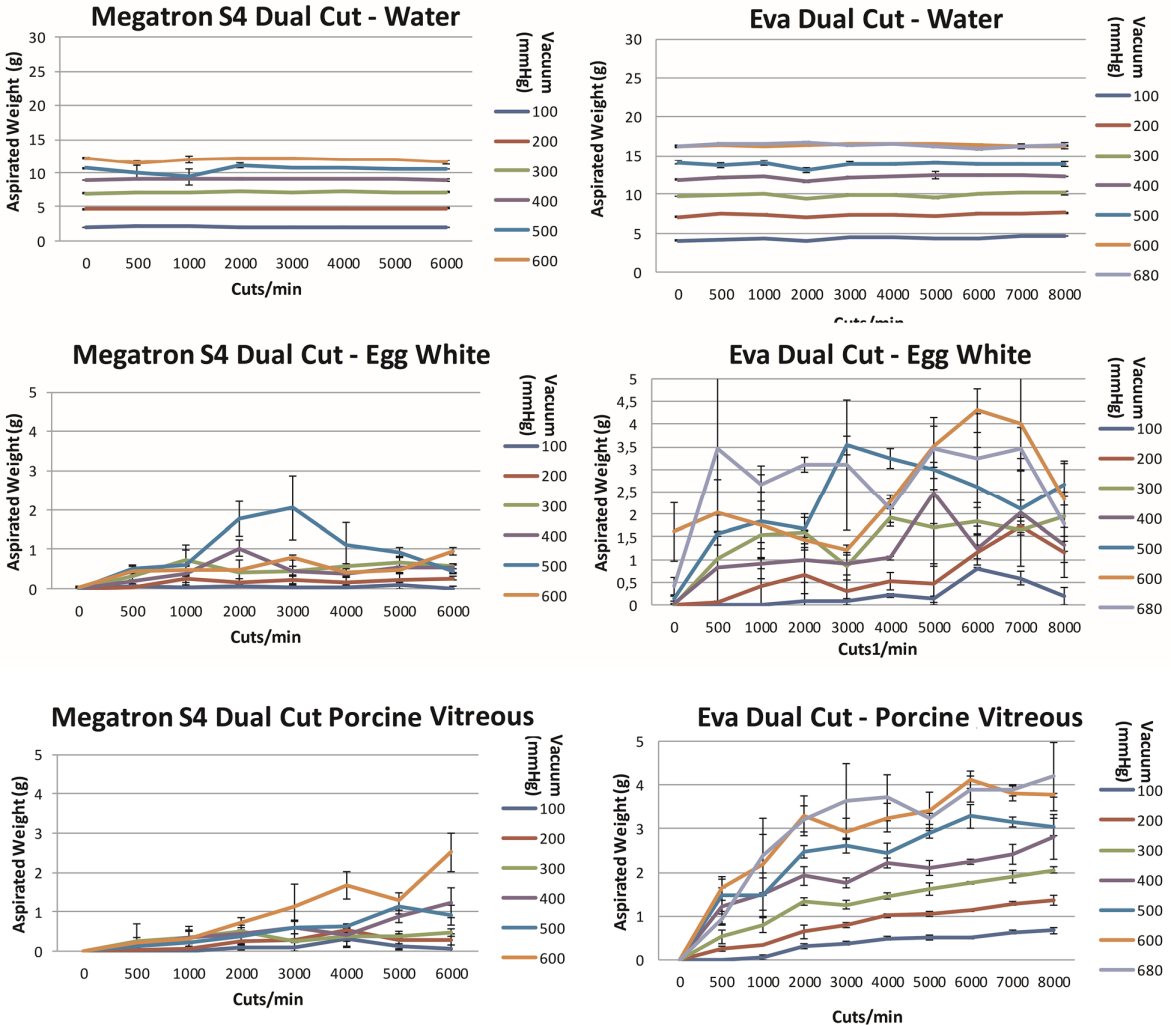
The aspiration of water (g), egg white and porcine vitreous as a function of cut rate and vacuum pressure for 23-gauge dual-cut vitrectors for the megaTRON S4^HPS^ and EVA^™^ vitrectomy systems

Comparing mono to dual-bladed probes, dual-bladed probes (Fig. 3) of megaTRON S4^HPS^ and EVA™ showed significantly higher (p < 0.0001) mass aspiration of water compared to its mono-beveled vitrectors (Fig. 2). The mean percentual difference at different cut rates was 45% for megaTRON S4^HPS^ and 79.7% for EVA™.

#### Pluronic gel

**Fig. 4:**
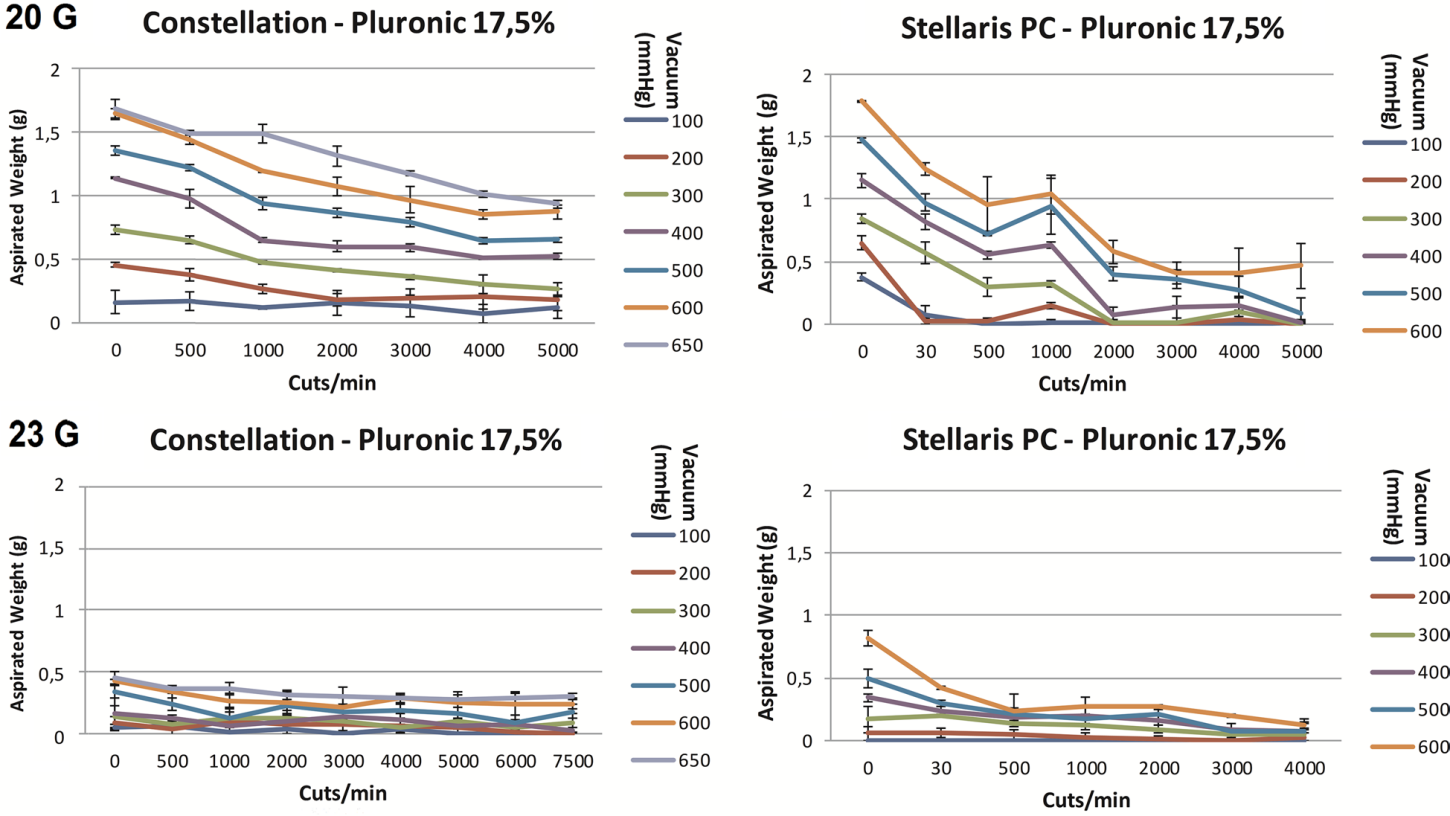
The aspiration of Pluronic gel (g) as a function of cut rate and vacuum pressure for 20- and 23-gauge vitrectomes for the Stellaris^®^ PC, Constellation^®^ Vision System, vitrectomy systems

Like water, the same relationship between cut rate vacuum and aspirated mass was detected when using Pluronic gel (Fig. 4). By considering its higher viscosity, less Pluronic gel was aspirated than water. Given the similarities between water and Pluronic gel regarding their homogeneity, further experiments were not performed on Pluronic gel.

#### Egg white

**Fig. 5:**
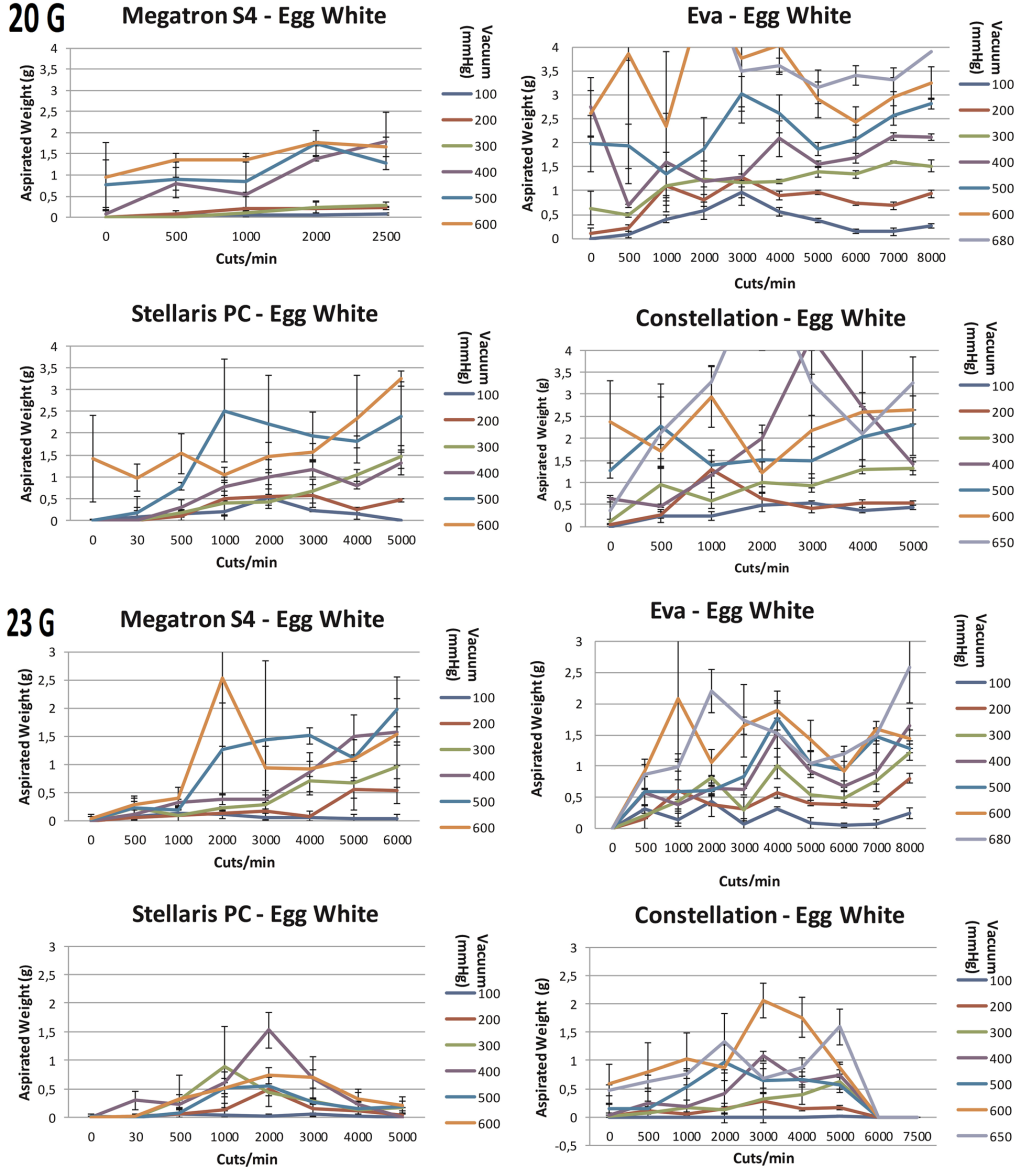
The aspiration of egg white (g) as a function of cut rate and vacuum pressure for 20- and 23-gauge vitrectomes for the vitrectomy systems Stellaris^®^ PC, Constellation^®^ Vision System, megaTRON S4^HPS^ and EVA^™^

For egg-white high standard deviations did not allow for the defining of significant differences between different vitrectomy systems using mono-bladed probes (Fig. 5). In addition, no significant differences for the aspirated mass at low vs high cut-rates were detected. Nevertheless, the area under the curve analysis (Fig. 7) indicates some decline or a slower increase of aspirated mass with increasing cut rates for some systems.

The megaTRON S4^HPS^ and EVA™ dual-bladed probes did not significantly increase nor decrease the weight of the aspirated mass compared to mono-bladed probes. This is based on a high standard deviation for some measurements (Fig. 3).

#### Porcine vitreous

**Fig. 6:**
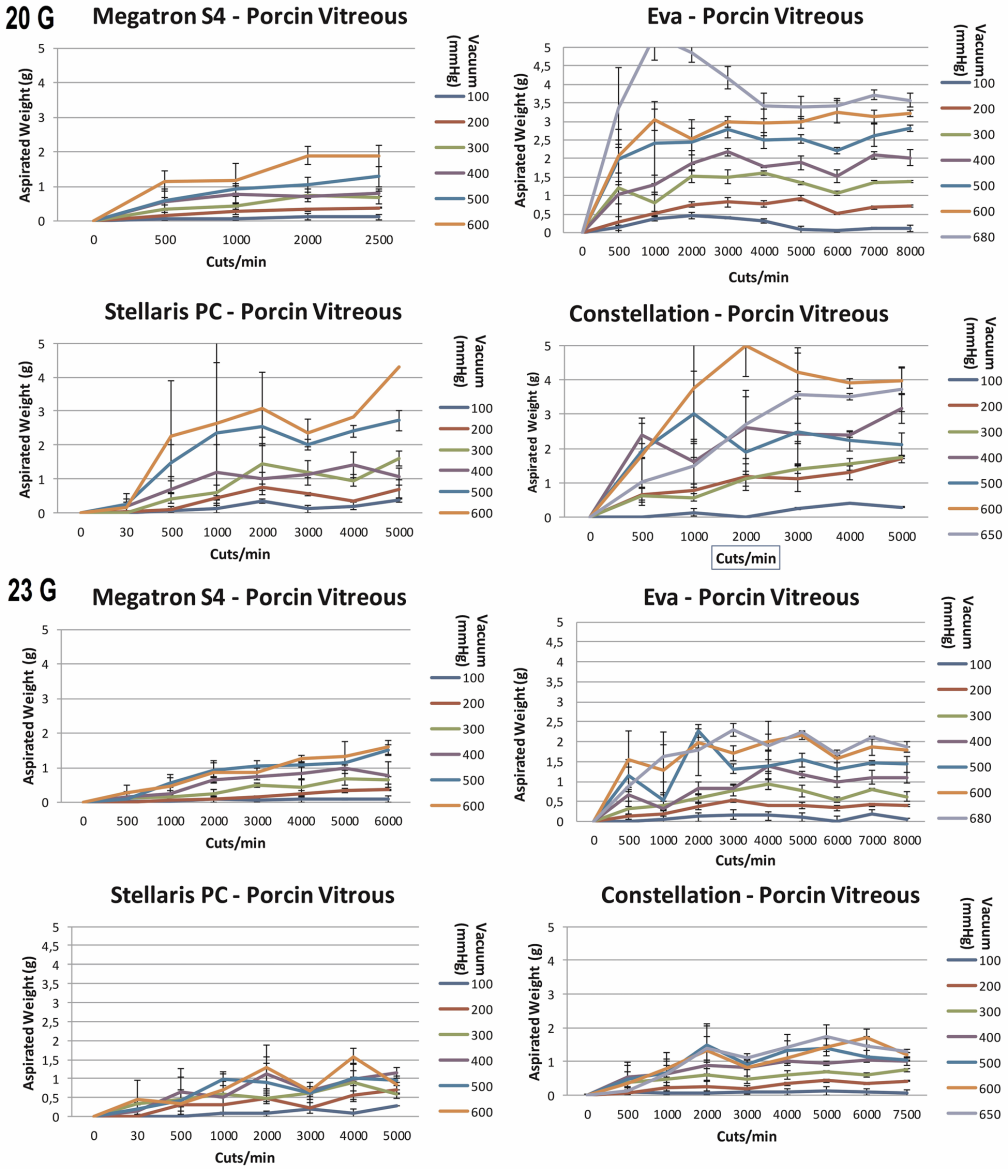
The aspiration of porcine vitreous (g) as a function of cut rate and vacuum pressure for 20- and 23-gauge vitrectomes for the vitrectomy systems Stellaris^®^ PC, Constellation^®^ Vision System, megaTRON S4^HPS^ and EVA^™^

**Fig. 7:**
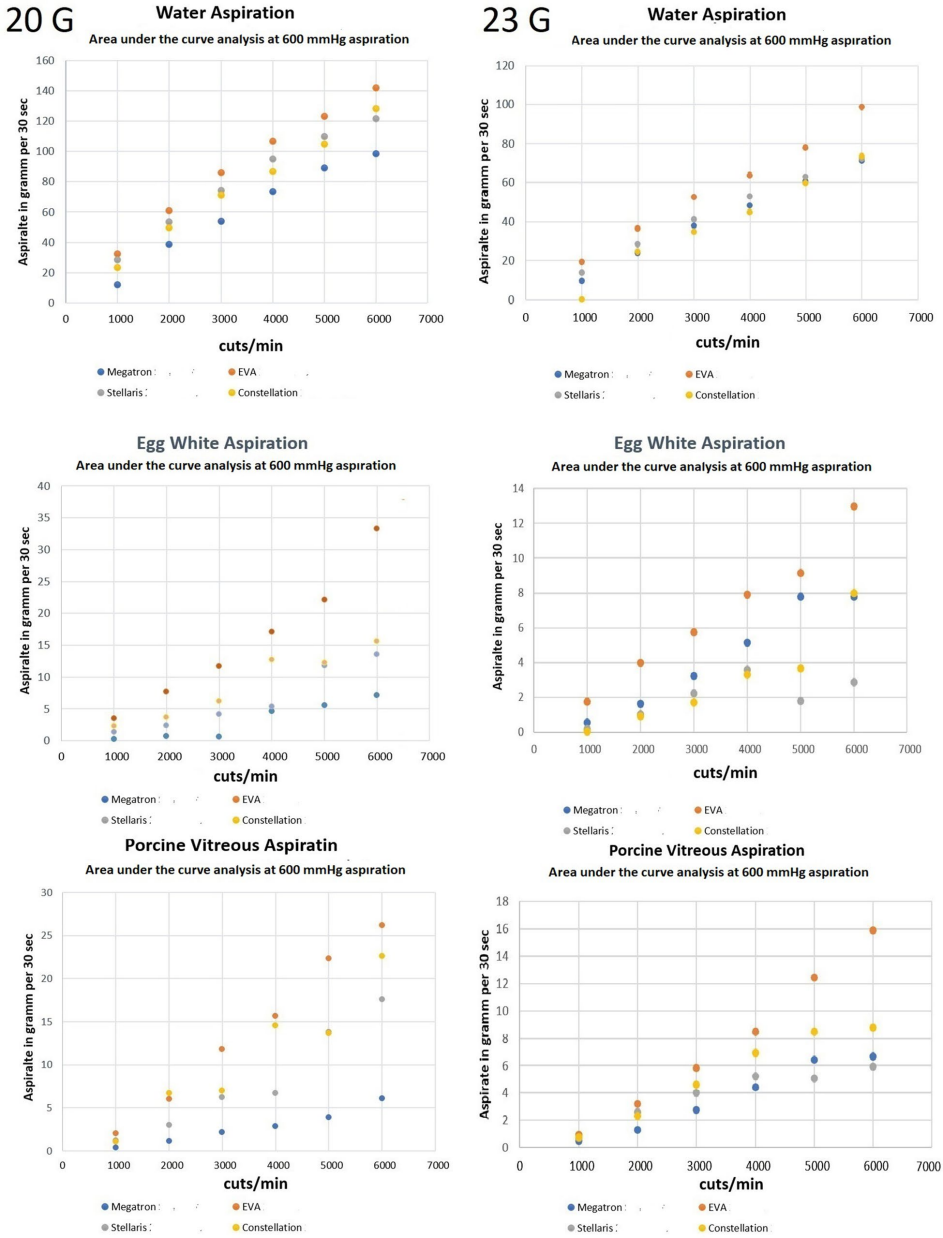
Area under the curve analysis of the aspiration of water (g), egg white and porcine vitreous as a function of cut rate and vacuum pressure for 20- and 23-gauge vitrectors for the vitrectomy systems Stellaris^®^ PC, Constellation^®^ Vision System, megaTRON S4^HPS^ and EVA™

Similarly to egg-white, the vitrectomy of porcine vitreous showed high standard deviations and therefore did not evidence significant differences between different vitrectomy systems using mono-bladed probes (Fig. 6). In addition, no significant differences for the aspirated mass at low vs high cut-rates were detected. Nevertheless, the area under the curve analysis (Fig. 7) indicates some decline or a slower increase of aspirated mass with increasing cut rates for some systems.

The megaTRON S4^HPS^ dual-cut vitrectors did not significantly alter the weight of the aspirated mass compared to its mono-bladed vitrector. Like with egg white, the dual cutter aspirated about the same amount of mass than the mono-bladed cutters at increasing cpm. For EVA™, at least at 6000 cpm, significantly more vitreous (p < 0.01) was aspirated using its dual-bladed compared to its mono-bladed probe. The mean (vacuum 100–680 mmHg) percentual difference at 6000 cpm was 117%.

### Area under the curve analysis

For a better comparison of the different systems, the area under the curve was analyzed with a fixed 600 mmHg vacuum pressure for all vitrectomy systems, while increasing cut rates from 0–6000 cpm (Fig. 7). In summary, the EVA™ system aspirated more of the different vitreous surrogates than the other systems.

### High speed video recordings

Blade movement during a duty cycle differs between various probes. Dual-bladed probes of the EVA™ and megaTRON S4^HPS^ systems showed constant vitreous aspiration compared to an oscillatory and enhanced vitreous movement of the mono-bladed probes of the Constellation^®^ Vision System, megaTRON S4^HPS^ and Stellaris^®^ PC systems. As examples, videos of the duty cycle of the dual cut Mach 2 (megaTRON S4^HPS^) compared to the UltraVit dual pneumatic cutter (Constellation^®^ Vision System) are shown (see Additional files 1, 2).

## Discussion

In this study we can demonstrate different models of vitreous and their characteristics during vitrectomy using current vitrectomy systems. It could be shown that water and biological gels substantially differ from one another. For water, we found with increasing cut-rates a significantly lower volume passing through the vitrector for most systems. Given the homogeneity in viscosity of this Newtonian fluid, a cutting process is not necessary. Therefore, higher cut rates adversely affect the vitreous removal, since the total time of a closed cutter during the duty cycle increases with higher cut rates. This effect has been confirmed by Diniz et al. using the Constellation^®^ system [17]. In their comparative study, cutter movements varied among the vitrectomes. The UltraVit dual pneumatic cutter of the Constellation^®^ system transported more fluid than classical spring return vitrectomes, considering its fast blade movement as confirmed by our high-speed video recordings of the vitrectome port opening. However, we could not confirm significant differences between different mono-bladed cutters. Therefore, we made an area under the curve analysis, which revealed the advantage of the UltraVit/ Constellation^®^ system, which was even more pronounced for EVA^™^ systems for all vitreous surrogates. One could speculate that the higher aspiration volume of the EVA^™^ system compared to their competitors is based on their novel pump system, which is neither a peristaltic nor a venturi pump. Their Vacuflow Valve Timing Intelligence (VTi) technology uses high-sensitivity pressure sensors and computer-controlled operating pistons, which generate a fast vacuum response and reduce undesirable flow turbulences [18]. In addition, its dual bladed probe showed significantly more water aspiration than its mono-bladed probe, supposedly due to the dual-bladed-cutter port being open for 92% of the duty cycle, according to information from DORC.

Egg white and porcine vitreous are semi-solid substances consisting of over 90% water and additional proteins. Both substances may obstruct the cutter opening [19]. While egg-white contains proteins such as albumins, mucoproteins and globulins, porcine vitreous proteins are mostly made up of hyaluronan-derived uronic acid and collagen [20], comparable to the collagen fibers and hyaluronic acid of human vitreous. Consequently, porcine vitreous shows a comparable viscosity and viscoelastic behavior to human vitreous [21, 22]. The inhomogeneity of these two vitreous surrogates might explain the high standard deviation seen in our experiments that did not allow us to perceive significant differences between various vitrectors and systems. Therefore, we used an area under the curve analysis, which indicated an increasing porcine vitreous/egg white removal in each machine, except for Stellaris PC, with increasing cut rates. As expected, 23-gauge vitrectors removed about half the amount of vitreous compared to 20-gauge ports. One reason for these lower flow rates is certainly based on Hagen–Poiseuille Law, which describes the pressure drop in an incompressible Newtonian fluid exhibiting laminar flow through a long cylindrical pipe of constant cross section. One could expect even lower amounts of vitreous being removed using 25 or 27 G devices. We excluded such devices from our study since not all producers had switched to a 25/27 gauge in 2016 when this study was performed.

The Dual-Cut cutters from Geuder and DORC showed some differences in performance. There was constant aspiration even at higher cut rates which, again, became significant only for EVA™ at 6000 cpm, presumably based on high standard deviations for the other experiments. Here, double-bladed vitrectors allow preforming forward and backward cutting of vitreous during blade movement, without complete closure of the cutter opening, which stands in contrast to standard guillotine-like cutters that have an increasing closure time at higher cut rates. This allows for a doubling of cut rates and might therefore increase the efficiency of vitrectomy even at higher cut rates. These results were confirmed by Pavlidis in a study of 80 patients using more novel two-dimensional (TDC) 25- and 27-gauge cutters (TDC, DORC International) for core vitrectomy. By using 25/27-gauge TDC rather than conventional cutters the overall core vitrectomy time could be reduced by 34–50% [23]. Another assumed disadvantage of mono-bladed probes is the safety issue, since complete port closure results in flow instability and fluid acceleration, which might cause retinal traction. This is consistent with our video imaging (Additional files 1, 2) where we did not find the typical oscillations of the vitreous, due to vitrector-port opening and closing of mono-bladed probes in comparison to dual-bladed probes. However, we could not demonstrate differences between vitreo-retinal interactions depending on the vitrectomy system used. Lima et al. describe another cutter design, a dual port cutter. Like dual-bladed cutters, dual-port cutters have the advantage of a faster vitreous aspiration than mono-bladed cutters [24]. Using dual-port cutters does not eliminate the safety concern, as their blades completely occlude the port during blade movement. However, retinal traction could very well be overcome by using dual-port cutters with smaller lumina in 25/27-G cutters, as discussed in a more recent review [25].

Overall, there are similarities in Newtonian fluid transport between single-cut cutters that can be ascribed to higher flow at higher vacuum pressures. There are lower flow rates for smaller cutters. The flow rates decrease more or less with increasing cut rates, depending on the cutter system (spring- pneumatic duty cycle). For vitreous surrogates such as egg white and porcine vitreous, we found an increase in aspirated mass, depending on the vacuum pressure and cutting movement. Different maxima were achieved with different machines. In most cases the vacuum pressure and cut rate correlate with the aspirated mass.

For surgeons, it is crucial to gain information about the oscillation of the retina in critical situations and the reproducibility of suction and cut rates to manipulate the vitreous without harming the retina. Dual-cutters change the apparition of the cutting systems by causing less vitreous movement. Further studies should address whether less vitreous movement as described here ultimately causes less retinal traction and thus helps to avert retinal tear formation.

## Supplementary Information

**Additional file 1.** Blade movement during a duty cycle of the dual-cut Mach2 cutter of the megaTRON S4^HPS^ system (Video 1) in comparison to the UltraVit dual pneumatic cutter of the Constellation Visual system (Video 2). Both videos were recorded at 5000 cpm and 200 mmHg aspiration pressure. The dual-cut probe of the megaTRON system showed less vitreous movement at the vitrector opening compared to the UltraVit probe of the Constellation.

**Additional file 2.** Blade movement during a duty cycle of the dual-cut Mach2 cutter of the megaTRON S4^HPS^ system (Video 1) in comparison to the UltraVit dual pneumatic cutter of the Constellation Visual system (Video 2). Both videos were recorded at 5000 cpm and 200 mmHg aspiration pressure. The dual-cut probe of the megaTRON system showed less vitreous movement at the vitrector opening compared to the UltraVit probe of the Constellation.

## Data Availability

The datasets analyzed during the current study are available from the corresponding author on reasonable request.
